# Letter to the editor regarding: ‘Effect of Tai Chi combined with visual-action-sensory rehabilitation therapies on cognitive function in acute ischemic stroke: a study protocol for a randomized controlled trial’

**DOI:** 10.1080/07853890.2026.2612788

**Published:** 2026-01-06

**Authors:** Fang Deng, Zhe Huang, Shuang Wu

**Affiliations:** a The Third School of Clinical Medicine (School of Rehabilitation Medicine), Zhejiang Chinese Medical University, Hangzhou, Zhejiang, China; b The Third Affiliated Hospital of Zhejiang, Chinese Medical University (Zhongshan Hospital of Zhejiang Province), Hangzhou, Zhejiang, China; c Research Institute of Tuina (Spinal disease), Zhejiang Chinese Medical University, Hangzhou, Zhejiang, China

Dear Editor

We read with interest the article ‘Effect of Tai Chi combined with visual-action-sensory rehabilitation therapies on cognitive function in acute ischemic stroke (AIS): a study protocol for a randomized controlled trial’ published in your journal [1]. The study offers valuable insights into the potential of Tai Chi for cognitive improvement in AIS patients, with implications for both clinical practice and research. However, several aspects warrant further discussion.

First, only the Last Observation Carried Forward (LOCF) was employed for data imputation despite an estimated 30% dropout rate, which cannot reflect the dynamic changes of cognitive function in AIS patients and may compromise the robustness of the findings. As the cognitive function of AIS patients changes dynamically with disease progression, LOCF overrelies on a single dropout observation and risks biased outcomes. We recommend conducting Little’s MCAR test first to identify missing data patterns, then combining multiple imputation and maximum likelihood estimation to minimize imputation-related biases [2]. Supplementary sensitivity analyses should also be performed to verify result consistency and enhance the robustness of study findings.

Second, the core assumptions of the selected statistical methods, repeated measures analysis of variance and linear mixed model, were not validated, which may weaken the credibility of statistical results. Critical assumptions including sphericity, homogeneity of variance and residual normality are essential for valid statistical inference; their absence may bias results and obscure true intervention effects. We suggest that subsequent studies test normality and homogeneity of variance for all continuous outcome variables [3], thereby providing evidence for the rational selection of statistical methods and enhancing the rigor of the research.

Third, concomitant medications were not dynamically monitored, potentially confounding intervention effects. Medications were not recorded during intervention and follow-up, nor incorporated as covariates into the statistical model. Concomitant medications may affect cognitive function in AIS patients [4]. Between-group differences or inadequate medication control may confound intervention outcomes. We recommend complete medication recording throughout the trial, incorporation of medications as covariates based on their cognitive impact, and sensitivity analyses stratified by medication status. This approach would better adjust for confounders and rigorously demonstrate intervention effects.

Fourth, the Minimum Clinically Important Difference (MCID) was excluded from outcome interpretation, with statistical significance as the sole evaluation criterion. These risks overestimating clinical relevance, as statistical differences do not always translate to clinically meaningful improvement. Although the study detailed assessment scales and intergroup statistical differences, it did not reference MCID thresholds or link score differences to clinical meaningfulness. We recommend MCID attainment analysis to clarify the proportion of patients with clinically significant benefits. This would enhance the clinical relevance of research conclusions and provide more reliable evidence for clinical decision-making.

In summary, Guan et al.’s study lays a valuable foundation for future research [1]. Implementing the above suggestions regarding statistical assumptions, missing data handling, medication adjustment, and clinical significance would help clarify causal mechanisms and provide more rigorous, clinically applicable evidence for cognitive rehabilitation in AIS patients.

## Data Availability

Data sharing does not apply to this article, as no data were created or analysed in this study.
